# Effects of an Orff Music Activity Intervention Program on the Ego-Resilience, Peer Relationships, Happiness, Interpersonal Care Awareness, Anxiety, and Stress of Children from Multicultural Families in Republic of Korea

**DOI:** 10.3390/healthcare11142095

**Published:** 2023-07-23

**Authors:** Su-Hee Kim, Sook Lee

**Affiliations:** 1 Department of Nursing, Baekseok University, Cheonan-si 31065, Republic of Korea; yani1505@naver.com; 2 Department of Nursing, Dankook University, Cheonan-si 31116, Republic of Korea

**Keywords:** child anxiety, child ego-resilience, child happiness, child peer relationships, child stress, interpersonal care awareness, interpersonal care nursing, Korean children, Orff music activities

## Abstract

This study developed an interpersonal care-based Orff music activity program for children from multicultural families in Republic of Korea. We measured its effects on ego-resilience, peer relationships, happiness, interpersonal care awareness, anxiety, and stress. The participants were 74 children from third to sixth grade attending 10 regional children’s centers in Cheonan-si, Chungcheongnam-do Province, Republic of Korea. They were randomly assigned to 36 experimental groups and 38 control groups. The experimental program was developed based on investigations of human care theory, expressions of care, and Orff music activities. The experiment was conducted twice a week for five weeks in 45 min sessions. Data were collected from June to September 2020. We used questionnaires on ego-resilience, peer relationships, happiness, and interpersonal care awareness to measure the effects, which were analyzed through frequency and descriptive statistics. Anxiety, physiological anxiety, acculturation stress, and physiological stress were measured using Behavior Detection VibraImage System Version 8.1 PRO (VIBRASYSTEM Ltd., Co., Seoul, Republic of Korea). The homogeneity of the variables across groups was examined using the χ² test, *t*-test, and Fisher’s exact test. The effects were examined using repeated measures variance analysis and independent *t*-tests. The results showed that the program had significant effects on ego-resilience, peer relationships, happiness, interpersonal care awareness, physiological anxiety, and physiological stress. The findings suggest that interpersonal care nursing can be effective for children from multicultural families, and the program can be used for intervention to improve children’s mental health.

## 1. Introduction

Multicultural families are generally composed of different nationalities, races, and cultures in which one parent is from another country. In 2019, the domestic marriage rate in Republic of Korea decreased by 7.2%, while the multicultural marriage rate increased by 4.0% over the previous year [1]. In terms of the composition of multicultural families, marriages between a Korean man and a foreign woman accounted for most at 69.3%, with 30.4% and 20.3% of female spouses being Vietnamese and Chinese, respectively [1].

The common characteristics of multicultural couples in Republic of Korea include the stress of adapting to a marriage with differences in cultural backgrounds, often a short dating period before marriage, and husbands who are aged. Because of conflicts and emotional struggles caused by the short adjustment period, the divorce rate among multicultural couples is 32.6% higher than that of Korean couples, and the average duration of marriage is 8.6 years shorter. Thus, although children in multicultural families are supported by nurturing foreign mothers as the primary caregivers, they are also exposed from an early age to a void in caregiving in the context of disintegrating families [1]. In 2019, the number of children from multicultural families increased by 5.9% compared with the previous year, while the total birth rate in Republic of Korea decreased by 7.4%, thereby increasing the proportion of children from multicultural families in the population [1]. The proportion of elementary school students from multicultural families was 76.1% in 2019, and preschoolers under age six accounted for 56.4% of all children in multicultural families. Thus, the number of school-aged children from multicultural families is expected to continue to increase in the future [2].

Children from multicultural families are raised in a dual cultural environment that often differs from that of their parents. They might also have difficulty learning Korean, resulting in poor academic ability. Furthermore, they are often discriminated against and bullied because of their different appearance, which increases stress and anxiety and lowers self-esteem. These can negatively affect school life adjustment, interpersonal caring awareness, and peer relationships, and can lead to identity confusion [3]. In particular, these children’s anxiety and stress may be high in terms of performance anxiety and the fear of being inadequate due to language, appearance, and biculturality. Furthermore, they may be reluctant to express their thoughts aloud, impeding their ability to form relationships and learn [4]. As such, children from multicultural families will need to strengthen their self-resilience to adapt to threatening environments [5]. Interactive psychological and emotional support programs need to be developed that can help school-aged children achieve higher self-resilience to cope with academic stress and improve their well-being and happiness [6].

Major factors influencing a child’s happiness include close parental relationships, strong attachments, and the feeling of being cared for through acts of love [7]. Happiness is a subjective emotion that when sufficiently satisfied can engender a more joyous life. Since childhood happiness can provide satisfying experiences throughout life, special attention should be devoted to children’s happiness. When children’s satisfaction with their parents is high, their peer relationships are better, and their mental health is stronger, leading to increased happiness [8].

A caring parent will help a child form positive relationships with others. Perceptions of care imply that children feel loved by their parents and that someone cares for them when they face difficulties [9]. A lack of parental care leads to unstable attachments and poor parental relationships, negatively affecting children’s relationships with others. Thus, the perception of care in childhood is important because it affects attachment in adulthood as well as fulfillment and contentment in various relationships, including marriage [10].

Although childcare can be provided in schools and the community, according to Leininger’s cross-cultural nursing theory, such care needs to consider cultural characteristics [11]. Women from other countries may feel isolated because of the lack of family and peer support during the birth and rearing of their children, which can be transmitted to their children as negative emotions [12]. Additionally, mothers’ lack of Korean language ability can affect their children’s language acquisition, making the children passive in expressing and asserting their thoughts among their peers. Peer relationships and acculturation stress in children have been reported as factors influencing ego-resilience [13] and happiness [14]. Existing support projects for children from multicultural families include language development support and multicultural awareness improvement projects; however, programs that raise awareness of how these children should receive care have not been developed [15,16].

According to Kim [17], considering the theory of human care, we need to develop programs that provide direct care to children and increase their awareness of the care they receive. Increased awareness of caring can help children change their negative emotions, regulate their emotions, recognize others’ emotions [18], and reduce anxiety and stress, thereby enhancing subjective happiness. Furthermore, considering the characteristics of children from multicultural families who may be passive in language expression and self-assertion, such programs can also improve communication with foreign mothers.

In general, group activities, rather than individual activities, are used to improve peer relationships. The aim of this experimental study was to develop such a program. Orff music activities convey social symbols or meanings by integrating music and rhythm into one through, for example, speaking, movement, dance, instrumental performance, role play, and improvisation, which are specific elements of expression. Gendron’s [19] notion of caring as a nonverbal communication method—which can be used by any care mediator in a community, even if the musical level is low—is widely regarded as the best technique for expressing theme, harmony, and melody elements, which are metaphorical expressions. Accordingly, we designed an interpersonal care-based Orff music activity program focusing on group activities for children from multicultural families and verified its effectiveness. This is a nursing intervention program that can improve children’s mental health and be utilized by caregivers in the community.

## 2. Materials and Methods

### 2.1. Participants

The experimental group comprised elementary school children in third through sixth grade from multicultural families in Republic of Korea. To determine the number of participants, we used G*POWER (G*POWER program 3.1.9.7, Heinrich Heine University, Düsseldorf, Germany) with reference to the effect size in Hong et al. [20]. Applying effect size = 0.25, alpha error = 0.05, power (1 − β) = 0.80, the two-tailed test, three-way repeated measures, and the correlation between repeated measurements of 0.50, the minimum number of participants required for the experimental or control group was 28. Considering a dropout rate of 30%, we recruited 74 participants, with 37 each in the experimental group and the control group. The control group comprised children from multicultural families in other regions and children who had not participated in other similar programs selected through convenience sampling. The specific selection criteria were children who could participate in music program activities without taking specific drugs for health reasons, excluding multicultural children who had immigrated midway. They needed to understand the purpose of the study and submit written consent from a legal representative to participate in the study.

### 2.2. Research Approach

We used a non-equivalent control group pre-/post-test design to evaluate the effect of the interpersonal care-based Orff music activity program on the mental health of children from multicultural families. Data were collected from June to September 2020. A pre-test was conducted one week before the experimental treatment and a post-test immediately after the program and two weeks after the experimental treatment, in both the experimental and control group. In the survey measurement, if the children faced difficulty in understanding the question or had problems with the measurement, a pre-trained local children’s center teacher informed them of the meaning and reviewed the question. The Behavior Detection VibraImage System Version 8.1 PRO (VIBRASYSTEM Ltd., Co., Seoul, Republic of Korea) and uBioMacpa pulse wave test methods (Biosense Creative, Seoul, Republic of Korea), which are physiological anxiety and stress measurement tests like a questionnaire, were also explained as non-invasive tests using the software.

We conducted the experimental treatment at four local children’s centers in Cheonan-si, Chungcheongnam-do Province, Republic of Korea, with the support of the teachers at the centers. After each session, we looked at the video recordings, analyzed the responses of the children, and created a video critique sheet and an activity sheet for the day. Moreover, after writing the critique and activity sheet for each session, a meeting was held with the head of the center and an activity assistant teacher, sharing activity videos with parents through social networking service communication, receiving feedback on the child’s condition and reaction, and reflecting these in the next session’s program activities.

To avoid researcher bias in conducting the program, all researchers were educated on the content and precautions of the program in a pre-meeting. Instrument settings and video installations were prepared 30 min before each program. Questionnaires and physiological measurement tests for effect verification were explained to help with data collection.

### 2.3. Orff Music Activity Program Development

We developed an Orff music activity program based on interpersonal care for children from multicultural families using the analysis, design, development, implementation, and evaluation (ADDIE) model [21]. “Analysis” refers to defining the learning content and determining what will be learned. “Design” is the process of specifying the teaching method based on the analysis results. “Development” involves actually creating programs to achieve the objectives identified in the analysis and design stages. “Implementation” refers to activities that use the program that has been created. The ”evaluation” stage involves determining the effectiveness and validity of the program; then, modifications are made on the basis of the evaluation.

#### 2.3.1. Analysis

To understand the characteristics and needs of children from multicultural families, we conducted a literature review of cultural care using Leininger’s sunrise model and in-depth reviews of health assessment tools to investigate the mental health status and program needs of children from multicultural families.

#### 2.3.2. Design

Goals for each program session were established based on the results of our analysis process. A logical study design, program operation method, and tools were selected to evaluate the program’s effectiveness. The structure of the interpersonal care-based Orff music activity program was divided into three areas—theme, harmony, and melody—following Gendron’s [19] metaphorical caring expression style through music. There were three sessions for each area. Each session comprised three stages: introduction, development, and consolidation. The last session comprised 10 integrated sessions. In the introduction, a song composed for the program was sung to announce the start of the program as a warmup through games and videos that fit the theme of the session. In the development stage, 10 interpersonal care techniques were applied in the theme area while performing activities, such as singing with Orff music, making ostinato rhythms, performing physical activities, playing musical instruments, and appreciating and playing musical instruments. Orff’s music activities deepen theme content through the physical activities of making rhythms, singing, playing instruments individually, and playing instruments in a group (ensembles) according to the title of each session. In the consolidation stage, the program ended by singing a “goodbye” song composed for the program, sharing feelings and promises for the next time they meet. To ensure smooth interaction, the number of children per group was limited to 10.

#### 2.3.3. Development

We created the program content by embedding the metaphorical caring expression method through Gendron’s music [19], based on the literature review and the specific needs of the research. The expressive care seen through the metaphor of music is composed of themes, harmony, and melody.

First, regarding themes, the theme was positive and warm, expressing care in a soft and gentle way. This reduces anxiety and stress in children, which are the goals of care, by opening their hearts to others and providing comfort. The Orff musical activities we used were singing, appreciation, and physical activity.

Second, the harmony aspect aimed to achieve agreement and synchronization through communication, namely harmony between caregiver and child. In our program, interpersonal intervention techniques centered on noticing were matched to achieve harmony between culture and personal characteristics, and between caregiver and child. The ensemble song was based on cultural issues, along with Orff’s techniques of singing, listening, and playing musical instruments by recognizing and understanding the cultural context of children from multicultural families. Through this, peer relationship, another goal of care, was improved through ensemble playing and performance.

Third, in the melody area, the care act between the care provider and child should be performed like a rhythm that occurs regularly and simultaneously in music. Paying attention to the child’s artistic experience in the melody area, interpersonal care techniques were matched with instilling hope (hoping) so that the caregiver could continuously and regularly engender hope in the participant. Orff’s music activities were designed to improve self-resilience and happiness, which are additional goals in caring for children in multicultural families, through singing, listening, playing instruments, playing in ensembles, and performing.

We validated the content of the program through an expert group. The expert group evaluating the program comprised one professor of psychiatric nursing, one lecturer in music education with a doctoral degree, two Orff instructors, one music therapist, and one mental health nurse, for a total of six experts.

The content validity of each item was evaluated on a four-point scale by applying the content validity index to the program goal, content and composition, operating time, application method, and appropriateness of the evaluation tool [22]. In addition, we adjusted the open-ended questions based on expert opinions (e.g., changing the title to be more friendly, incorporating various traditional percussion instruments). We also made changes to the amount of content according to the operating time (too much activity in the allocated time), the development process (e.g., rhythm making, physical activity, singing, playing musical instruments, ensemble/ensemble, improvisation), the elements that reflected culture (songs, dances, physical activities, and traditional musical instruments were more actively used and incorporated), and the complement of composed songs (harmony code changes in the “hello” and “goodbye” songs, foreign traditional songs in composed ensembles, and the use of more pentatonic scale elements based on folk songs).

In a preliminary study, an actual program modified based on expert validity was simulated in May 2020 with 10 children from multicultural families who had no experience participating in music activity programs. The program was modified by collecting opinions from the children on content, overall operating time, and supplementary methods. Furthermore, a mental health nurse, a music therapy expert, and three caregivers from the center were asked to observe the class and assess what needed to be added. The program was revised and supplemented through the final review and preliminary expert opinions. Subsequently, our Orff music activity program based on personal care was completed.

#### 2.3.4. Implementation

We conducted our program twice a week for five weeks in five regional children’s centers from 8 June to 16 September 2020, for twice a week, for 45 min per session, for five weeks, for a total of 10 sessions. Groups were created for each center, with a maximum of 10 participants per group. After each session, we held a meeting with the center director to manage the program, respond to participants, and share opinions with parents.

#### 2.3.5. Evaluation

Immediately after the end of the interpersonal care-based Orff music activity program, we distributed and collected project post-mortem questionnaires. Furthermore, we conducted post-tests five and seven weeks after the end of the program. Moreover, we measured physiological anxiety using Behavior Detection VibraImage System Version 8.1 PRO (VIBRASYSTEM Ltd., Co., Seoul, Republic of Korea), taking precautions such as advising subjects not to laugh, move, lean back on the chair, or wear jewelry. The measurement location was a quiet space, and the measurement distance was adjusted. Physiological stress refers to a score measured by the uBioMacpa V70 (Biosense Creative, Seoul, Republic of Korea) for heart-rate variability. Precautions for using this to measure stress indicate that the participant should refrain from heavy exercise, eating, showering, or coffee for one hour before the measurement, and refrain from talking, heavy movement, breathing, sighing, or thinking during the test. We cautioned the children accordingly and the participants were refrained from engaging in behaviors that affect the autonomic nervous system. Considering that even a slight movement can make a difference in the measurement, the children were allowed to rest for 10 min, and then their index fingers were placed on the device in a comfortable position and measured for 2 min and 30 s.

### 2.4. Ethics Statement

This study was reviewed and approved (registration no: DKU 2018-11-010-002) by the institutional review board (IRB) of Dankook University, Cheonan-si, Chungcheongnam-do Province, Republic of Korea. When recruiting participants, the children and their legal representatives were notified of the purpose and procedure of the study, anonymity and confidentiality of data processing, withdrawal from the study was explained, and written consent was obtained. A small gift was provided to all participants in the study.

### 2.5. Data Analysis

We used SPSS (version 23.0; IBM Corp., Armonk, NY, USA) for data analysis. The general characteristics of the participants were analyzed by frequency, percentage, mean, and standard deviation. The general characteristics and the homogeneity of dependent variables were verified through the χ² test, Fisher’s exact test, and independent *t*-tests. We evaluated the effect of the Orff music activity program based on personal care using the repeated measure variance analysis. Furthermore, we analyzed Cronbach’s alpha to assess the reliability of the tool, with a statistical significance set at 0.05.

## 3. Results

### 3.1. Effectiveness of the Orff Music Activity Program Focused on Interpersonal Caring Awareness

#### 3.1.1. Homogeneity of the Study Group

The homogeneity test checked the general characteristics of the experimental group and the control group by grade, gender, age, educational level, occupation, mother’s communication language, as well as her ability to speak Korean and her nationality, and dominant caregiver, house income level, and school performance. There were no significant differences between the two groups for any of the variables (*p* > 0.05), indicating that they were homogeneous (Table 1).

There were no statistically significant differences in ego-resilience, peer relationship, happiness, interpersonal caring awareness, anxiety, physiological anxiety, acculturation stress, and physiological stress between the experimental group and the control group (*p* > 0.05). Table 2 shows the prior homogeneity between the two groups.

#### 3.1.2. Hypothesis Test

As shown in Table 3, statistically significant differences were found in ego-resilience between the groups (F = 29.360, *p* < 0.001), time (F = 189.392, *p* < 0.001), and the interaction of the two (group × time) (F = 18.728, *p* < 0.001). Regarding the difference between the experimental and control groups at each period, after five weeks, the experimental group and control group scored 3.36 and 2.49 points, respectively; thus, ego-resilience was significantly higher in the experimental group (t = 7.475, *p* < 0.001). After seven weeks, the experimental group scored 3.16 points and the control group 2.60 points; again, the experimental group was significantly higher (t = 4.483, *p* < 0.001).

Peer relationships also showed statistically significant differences between the groups (F = 9.743, *p* = 0.003), time (F = 25.074, *p* < 0.001), and interactions (F = 10.898, *p* < 0.001). Regarding the difference between the experimental group and the control group at each time period, after five weeks the experimental group scored 2.41 points and the control group 1.99 points, indicating a significantly higher difference in the experimental group (t = 3.419, *p* < 0.001). After seven weeks, the experimental group scored 2.59 points and the control group scored 2.01 points; again, the experimental group was significantly higher (t = 4.889, *p* < 0.001).

There were statistically significant differences in happiness between the groups (F = 10.405, *p* < 0.001), time (F = 14.414, *p* < 0.001), and interactions (F = 5.538, *p* = 0.020). Regarding the difference between the experimental group and the control group at each period, after five weeks, the experimental group scored 2.81 points and the control group 2.27 points, thus showing a significantly higher score in the experimental group (t = 5.199, *p* < 0.001). After seven weeks, at 2.36 points the experimental group scored higher than the control group at 2.27 points, but the difference was not statistically significant (*p* = 0.354).

There were statistically significant differences between groups (F = 6.213, *p* = 0.015), time (F = 8.910, *p* < 0.001), and interactions (F = 13.706, *p* < 0.001) in total interpersonal care awareness. Regarding the difference between the experimental group and the control group at each period, after five weeks, the experimental group scored 3.88 points and the control group 3.26 points; thus, interpersonal care awareness was significantly higher in the experimental group (t = 3.918, *p* < 0.001). After seven weeks, the experimental group continued to score significantly higher at 3.81 points than the 3.26 points in the control group; thus, the effect of the program continued (t = 3.138, *p* = 0.002).

There was a significant difference in anxiety over time (F = 6.089, *p* = 0.003). However, there were no statistically significant differences between the groups or interactions (*p* > 0.05). Regarding the difference between the experimental group and control group at each time period, the 1.46 points each in the experimental group and control group showed no statistically significant difference after five weeks (*p* > 0.05). After seven weeks, the experimental group scored 1.40 points and the control group scored 1.46 points; thus, the score was slightly higher in the control group than in the experimental group, but the difference was not statistically significant (*p* > 0.05).

There was no statistically significant difference in physiological anxiety between the groups and time (*p* > 0.05); however, there was a statistically significant difference in interactions (F = 3.298, *p* = 0.040). Regarding the difference between the experimental group and control group at each time period, after five weeks, the experimental group scored 58.57 points and the control group 63.05 points; thus, physiological anxiety interactions were higher in the control group than in the experimental group, though this was not statistically significant (*p* > 0.05). After seven weeks, the experimental group scored 60.91 points and the control group 64.35 points; again, the control group scored higher, showing a statistically significant difference, indicating that the effect of the program was delayed (t = −2.160, *p* = 0.034).

There was a statistically significant difference in acculturative stress between the groups (F = 4.231, *p* = 0.043) but no statistically significant difference between time and interactions (*p* > 0.05). Regarding the difference between the experimental group and the control group at each period, after five weeks, the experimental group scored 1.19 points and the control group 1.37 points, showing a statistically significantly lower score in the experimental group (t = −3.470, *p* < 0.001). After seven weeks, the control group scored 1.27 points and the experimental group 1.15 points, showing a statistically significant difference (t = −2.457, *p* = 0.016).

There were no statistically significant differences in physiological stress overall between the groups, but there was a statistically significant difference between time (F = 5.086, *p* = 0.005) and interactions (F = 9.560, *p* < 0.001). Regarding the difference between the experimental group and control group at each period, the experimental group scored 27.87 points and the control group 29.13 points after five weeks; thus, the difference was lower in the experimental group than in the control group but not statistically significant (*p* > 0.05). Even after seven weeks, the experimental group scored 25.96 points and the control group 29.00 points, with no statistically significant difference (*p* > 0.05).

## 4. Discussion

We developed a program focusing on the interpersonal care technique of the human care model. We confirmed the program’s effectiveness by examining interpersonal care awareness, ego-resilience, peer relationships, anxiety and stress, and happiness. After participating in the program, interpersonal care awareness scores increased significantly in the experimental group, and the program’s effect was maintained even after two weeks. Prior to studies of upper elementary school children [9,23], workers [24], nursing students [25], and nurses [26,27] have also found that interpersonal care awareness increases after intervention programs.

In our study, the Orff music activity program enabled the children to express their emotions through music, recognize the feelings and situations of others, and acknowledge that they had been cared for and emotionally supported. Through the Orff music activities, we provided opportunities to actively understand other children through songs, music, and dances from the mother’s country while increasing interactions with parents. As such, the child’s sense of being taken care of increased. Regarding specific interpersonal care behaviors, awareness of participating, comforting, hoping, sharing, active listening, being a companion, and noticing increased significantly in the experimental group compared with the control group.

The ego-resilience score also increased significantly in the experimental group, and this effect was maintained even after two weeks. This supports the findings of Park and Kim [28]. Ego-resilience can be divided into five components: vitality, emotional control, interpersonal relationships, self-acceptance, and optimism. In Lee’s [29] study, art activities produced significant changes in children from multicultural families, but the lowest scores were in emotional control. Park and Kim [28] likewise found no significant difference in emotion regulation. The reasons for this include difficulty expressing and controlling emotions in the case of children from broken and multicultural families, as well as an inability to express their emotions appropriately. We believe the significant improvement in ego-resilience scores in our study can be attributed to the techniques of comforting and noticing, along with music activities, which were developed to induce hope—a caring act that increases ego-resilience.

Taylor et al. [30] noted that ego-resilience helps children express empathy, through which they can express their own emotions while also understanding the emotions of others. Our interpersonal care-based music activity program recognized the unique emotional and psychological states of multicultural children. We found that parental attachment and elasticity increased through the process of discussing feelings with friends, expressing feelings in general, listening to each other, and fostering understanding. This was all achieved through our program, which was conducted in large groups with support from a mediator. Furthermore, movement activity, imitation, and improvisational ensembles, which are Orff musical activity techniques, helped increase flexibility through activities that improved mutual recognition and understanding. This supports the findings of previous studies focused on the usefulness of group experiences [31]. Ultimately, our program had significant and positive effects on the ego-resilience of children from multicultural families.

We also found that the peer relationship score increased significantly in the experimental group, and this effect was maintained even after two weeks. Lee [32] found that, in the context of a percussion ensemble, peer relationships improved through mutual exchange, empathy, and consideration, based on an understanding and acceptance of others. Akbari et al. [33] found that participants experienced emotional support through music therapy based on improvisation, singing, appreciation, discussion, and other activities; this helped achieve therapeutic goals by releasing emotional regulation and forming cooperative relationships. Moreover, music can be useful for people who have difficulty relating to or interacting with strangers via nonverbal communication; it is considered effective in cases of autism, learning disabilities, and attachment disorders. In our study, such benefits arose from the Orff instruments being played together, along with ensemble performances [33]. Previous studies have also shown that empathy is a factor that directly affects peer relationships in school-aged children [34]. Yeom [35] found that among children from multicultural families, parents’ warm and accepting attitudes had positive effects on peer relationships. Empathy corresponds to noticing, feeling, and recognizing another person’s feelings; if parents, teachers, and families provide care through praise, acceptance, and encouragement, children’s peer relationships can be further improved.

In this study, the happiness score increased significantly in the experimental group. This effect increased immediately after the program but decreased two weeks later. These results align with those of Kong and Chae [36] and Pandya and Yoga [37] with regard to measuring happiness immediately after an intervention. Kong and Chae [36] found that their intervention produced positive scores in the subdomains of enjoyable life, immersive life, and meaningful life. These effects continued after the intervention ended, which was attributed to a gratitude diary record and a happiness experience activity. Yim [38] also noted that a gratitude disposition can be developed and optimism nurtured by learning through gratitude diary writing and self-directed tasks. In our study, however, increased social distancing and changes in when children went to school due to the second wave of COVID-19 occurring between the post-test immediately after the program and that two weeks later might have had an impact. This could have lowered the maintenance of happiness. Going forward, we need to find a way to continuously maintain the happiness that children feel after their interactions in the program.

Our program did not significantly reduce anxiety in children from multicultural families. Goldbeck and Ellerkamp [39] applied music therapy combined with cognitive behavioral therapy to children diagnosed with anxiety disorders. Their results showed a significant reduction in anxiety lasting four months. Lee [40] found that administering the Orff music program to juvenile offenders reduced aggression, anxiety, tension, and sense of inferiority, among the subitems of emotional instability. Thus, music can be used as a tool to reduce emotional instability when expressing one’s emotions, and it can be an appropriate means of expression for boys. We believe the lack of significantly reduced anxiety in our study could be attributable to the weaker emotional and psychological states of multicultural children, their delayed linguistic development caused by dual-culture parenting, and their sense of anxiety owing to prejudice against foreigners and bullying. Additionally, the mediation time was likely insufficient for resolving these issues. However, our program did significantly reduce physiological anxiety scores. VibraImage offers an objective measurement that reflects all body organ characteristics up to 100% with a non-contact device, such as a digital camera. Kim and Kim [41] found significantly lowered anxiety and tension scores measured using VibraImage post intervention. Although there was no change in anxiety scores according to our questionnaire measurements, physiologically measured anxiety scores decreased. In this respect, we confirmed that there was a difference between the states of physiological and cognitive anxiety. Further research is needed in this regard.

In particular, regarding the anxiety measurement method, VibraImage can highlight a more subtle difference through a non-significant result, although anxiety was lower than the average in the questionnaire. Kim and Lee [42] measured loneliness and self-esteem in children from single-parent families, but the subjective questionnaires did not show statistically significant differences. Yet, when measuring heart-rate variability using changes in the autonomic nervous system, which is a physiological indicator of a stress response, there were statistically significant differences in sympathetic nervous system changes, parasympathetic nervous system changes, and the ratio of sympathetic and parasympathetic nervous system activities. This supports the results of our study.

Furthermore, our interpersonal care-based Orff music activity program did not significantly reduce the acculturation stress scores of these children from multicultural families. This differs from Ko [43], who reported that acculturation stress was reduced as a result of music therapy centered on traditional nursery rhymes. Moreover, Yoon and Kang [44] found a statistically significant difference in acculturation stress based on a song psychotherapy program. By contrast, Yoo [45] administered music therapy centered on percussion instrument performance to Mongolian middle school students in Korea; the before-and-after comparison did not show a significant reduction in acculturation stress. Meanwhile, our interpersonal care-based Orff music activity program showed partially reduced physiological stress scores for children from multicultural families. Physiological stress is an index measured using uBioMacpa. A previous study showed that music therapy centered on percussion instrument performance lowered the stress scores of elementary school children, which differed from the type of performance used by Ko [43]. Keum [46] also supported the idea that listening to traditional Korean music significantly reduced the stress index of adolescents.

Our findings have the following implications for nursing. First, in the field of mental health nursing education, the educational content should be expanded to promote mental health nursing intervention in the community. Second, regarding nursing research, along with the empirical verification of the human care model [17], the variables of personal care techniques should be expanded, and the target of the model should include children. Third, existing nursing music activity therapy should be combined with interpersonal care techniques to expand the practical nursing interventions available to psychiatric nurses.

Regarding this study’s limitations, children in the “mixed” experimental group (children from multicultural families and children with Korean parents) might have had a higher level of awareness of being subject to increased care, which could have also intensified the sense of separateness. However, we did not conduct classes in a “mixed” group. Future research can focus on analyzing “mixed” groups together. In addition, because of COVID-19, a foreign mother who provided direct care to a child could not directly participate and instead shared activity videos on social media to check the child’s condition and reaction. Moreover, the period of residence in Republic of Korea for foreign mothers was not considered. Future research should confirm this study’s results using a larger number of subjects and regions. We, therefore, recommend a repeated-measurement study to generalize the results and a follow-up study considering the child’s growth and development.

## 5. Conclusions

We confirmed that an interpersonal care-based Orff music activity program improved ego-resilience, peer relationships, happiness, interpersonal care awareness, physiological anxiety, and physiological stress in children from multicultural families. Thus, we recommend that this program be expanded for use with more children from multicultural families. The program can be used as a form of nursing intervention to maintain and promote children’s mental health. Finally, based on our results, further research that assesses the effectiveness of our program for other children from vulnerable groups should be conducted.

## Figures and Tables

**Table 1:** Homogeneity test of general characteristics (*n* = 74).

Characteristic		Categories	Experimental (*n* = 36)	Control (*n* = 38)	χ² or *t*	*p*
Grade (elementary school)		3rd	13 (36.2)	16 (42.2)	5.997	0.307
		4th	7 (19.4)	9 (23.7)		
		5th	12 (33.3)	6 (15.8)		
		6th	4 (11.1)	7 (18.4)		
Sex		Male	18 (50.0)	23 (60.5)	0.829	0.363
		Female	18 (50.0)	15 (39.5)		
Age (in years)	Father	≤30–39	2 (5.6)	2 (5.3)	0.895	0.639
		40–49	18 (50.0)	15 (39.5)		
		≥50–59	16 (44.4)	21 (55.3)		
	Mother	≤30–39	31 (86.1)	27 (71.1)	3.367	0.186
		40–49	5 (13.9)	9 (23.7)		
		≥50–59	0 (0)	2 (5.3)		
Educational level	Father	Middle school	6 (16.7)	5 (13.2)	0.442	0.931
		High school	27 (75.0)	29 (76.3)		
		≥College	3 (8.4)	4 (10.6)		
	Mother	Middle school	13 (36.1)	10 (26.3)	3.287	0.349
		High school	16 (44.4)	22 (57.9)		
		≥College	7 (19.5)	6 (15.8)		
Occupation	Father	White collar	7 (19.4)	4 (10.5)	8.844	0.264
		Self-employment	0 (0)	1 (2.6)		
		Agriculture	6 (16.7)	10 (26.3)		
		Factory worker	11 (30.6)	8 (21.1)		
		Serving	1 (2.8)	0 (0)		
		None	9 (24.9)	7 (18.4)		
		Others	2 (5.6)	8 (21.1)		
	Mother	White collar	4 (11.1)	4 (10.5)	6.570	0.475
		Self-employment	5 (13.9)	1 (2.5)		
		Agriculture	1 (2.8)	4 (10.5)		
		Factory worker	6 (16.7)	9 (23.7)		
		Serving	6 (16.7)	3 (7.9)		
		None	11 (30.5)	12 (31.7)		
		Others	3 (8.3)	5 (13.2)		
Mother’s communication language		Korean	24 (66.7)	28 (73.7)	0.436	0.509
		Korean + half of mother country language	12 (33.3)	10 (26.3)		
Mother	Korean ability	Speaking	3.97 ± 0.971	3.76 ± 1.076	0.876	0.384
		Listening	4.06 ± 1.145	3.89 ± 1.034	0.635	0.528
		Reading	3.67 ± 1.146	3.84 ± 1.053	−0.686	0.495
		Writing	3.44 ± 1.297	3.68 ± 1.248	−0.776	0.440
	Nationality	China	4 (11.1)	2 (5.3)	3.415	0.491
		Vietnam	27 (75.0)	27 (71.1)		
		Philippines	4 (11.1)	4 (10.5)		
		Cambodia	1 (2.8)	4 (10.5)		
		Others	0 (0)	0 (0)		
Dominant caregiver		Parents	16 (44.4)	24 (63.2)	10.554	0.061
		Grandparents	3 (8.3)	1 (2.6)		
		School teacher	6 (16.7)	0 (0)		
		Assistant teacher	1 (2.8)	1 (2.6)		
		Center teacher	10 (27.8)	10 (26.3)		
		Others	0 (0)	2 (5.3)		
House income level		Low	2 (5.6)	1 (2.6)	2.673	0.263
		Middle	15 (41.7)	10 (26.3)		
		High	19 (52.8)	27 (71.1)		
School performance		Low	11 (30.6)	14 (36.8)	2.010	0.366
		Middle	11 (30.6)	15 (39.5)		
		High	14 (38.9)	9 (23.7)		
Total			36 (100)	38 (100)		

Data are expressed as *n* (%) or mean ± standard deviation. Assessed by chi-square or independent *t*-test.

**Table 2:** Homogeneity of the dependent variables.

Variables		Experimental (*n* = 36)	Control (*n* = 38)	*t*	*p*
Ego-resilience		1.72 ± 0.56	1.70 ± 0.47	0.169	0.867
Peer relationship		1.90 ± 0.58	1.87 ± 0.64	0.180	0.858
Happiness		2.31 ± 0.34	2.33 ± 0.61	−0.211	0.834
Interpersonal caring awareness	Total	3.26 ± 0.73	3.32 ± 0.76	−0.378	0.706
Anxiety	Anxiety	1.62 ± 0.45	1.50 ± 0.45	1.087	0.281
	Physiological anxiety	64.40 ± 6.07	62.99 ± 7.02	0.921	0.360
Stress	Acculturation stress	1.25 ± 0.35	1.27 ± 0.48	−0.242	0.809
	Physiological stress	31.51 ± 11.23	28.33 ± 9.39	0.921	0.360

Data are expressed as mean ± standard deviation. Assessed by independent *t*-test.

**Table 3:** Comparison of the variables between groups (*n* = 74).

Variable	Groups	Experimental (*n* = 36)	Control (*n* = 38)	*t*	*p*	Source	*F*	*p*
Ego-resilience	Pre	1.72 ± 0.56	1.70 ± 0.47	0.169	0.867	Group	29.360	<0.001 ***
	Post 1 (5 wks)	3.36 ± 0.34	2.49 ± 0.62	7.475	<0.001 ***	Time	189.392	<0.001 ***
	Post 2 (7 wks)	3.16 ± 0.49	2.60 ± 0.59	4.483	<0.001 ***	Group × Time	18.728	<0.001 ***
Peer relationship	Pre	1.90 ± 0.58	1.87 ± 0.64	0.180	0.858	Group	9.743	0.003 **
	Post 1 (5 wks)	2.41 ± 0.32	1.99 ± 0.68	3.419	<0.001 ***	Time	25.074	<0.001 ***
	Post 2 (7 wks)	2.59 ± 0.26	2.01 ± 0.68	4.889	<0.001 ***	Group × Time	10.898	<0.001 ***
Happiness	Pre	2.31 ± 0.34	2.33 ± 0.61	−0.211	0.834	Group	10.405	<0.001 ***
	Post 1 (5 wks)	2.81 ± 0.44	2.27 ± 0.46	5.199	<0.001 ***	Time	14.414	<0.001 ***
	Post 2 (7 wks)	2.36 ± 0.44	2.27 ± 0.46	0.933	0.354	Group × Time	5.538	0.020 *
Interpersonal caring awareness	Pre	3.26 ± 0.73	3.32 ± 0.76	−0.378	0.706	Group	6.213 *	0.015 *
	Post 1 (5 wks)	3.88 ± 0.48	3.26 ± 0.84	3.918	<0.001 ***	Time	8.910	<0.001 ***
	Post 2 (7 wks)	3.81 ± 0.67	3.26 ± 0.84	3.138	0.002 **	Group × Time	13.706	<0.001 ***
Anxiety	Pre	1.62 ± 0.45	1.50 ± 0.45	1.087	0.281	Group	0.048	0.826
	Post 1 (5 wks)	1.46 ± 0.40	1.46 ± 0.39	−0.016	0.988	Time	6.089	0.003 **
	Post 2 (7 wks)	1.40 ± 0.42	1.46 ± 0.39	−0.593	0.555	Group × Time	2.480	0.087
Physiological anxiety	Pre	64.40 ± 6.07	62.99 ± 7.02	0.921	0.360	Group	2.470	0.120
	Post 1 (5 wks)	58.57 ± 11.44	63.05 ± 11.03	−1.714	0.091	Time	2.845	0.061
	Post 2 (7 wks)	60.91 ± 7.56	64.35 ± 6.09	−2.160	0.034 *	Group × Time	3.298	0.040 *
Acculturation stress	Pre	1.25 ± 0.35	1.27 ± 0.48	−0.242	0.809	Group	4.231	0.043 *
	Post 1 (5 wks)	1.19 ± 0.23	1.37 ± 0.23	−3.470	<0.001 ***	Time	1.415	0.246
	Post 2 (7 wks)	1.15 ± 0.17	1.27 ± 0.25	−2.457	0.016 *	Group × Time	2.067	0.130
Physiological stress	Pre	31.51 ± 11.23	28.33 ± 9.39	1.327	0.189	Group	0.036	0.851
	Post 1 (5 wks)	27.87 ± 7.16	29.13 ± 10.06	−0.618	0.539	Time	5.586	0.005 **
	Post 2 (7 wks)	25.96 ± 6.75	29.00 ± 9.43	−1.583	0.118	Group × Time	9.560	<0.001 ***

Data are expressed as mean ± standard deviation. * *p* < 0.05; ** *p* < 0.01; *** *p* < 0.001, assessed by repeated measures analysis of variance and independent *t*-test.

## Data Availability

The data presented in this study are available upon request from the authors. Some variables were restricted to preserve the anonymity of study participants.
